# The relationship between systemic immune inflammation index and survival in patients with metastatic renal cell carcinomatreated withtyrosine kinase inhibitors

**DOI:** 10.1038/s41598-022-20056-3

**Published:** 2022-10-03

**Authors:** Kadriye Bir Yücel, Emre Yekedüz, Serdar Karakaya, Deniz Tural, İsmail Ertürk, Cihan Erol, Özlem Ercelep, Nihan Şentürk Öztaş, Çağatay Arslan, Gökhan Uçar, Ahmet Küçükarda, Özlem Nuray Sever, Saadettin Kılıçkap, Orçun Can, Satı Coşkun Yazgan, Berna Öksüzoğlu, Nuri Karadurmuş, Mehmet Ali Şendur, Yüksel Ürün

**Affiliations:** 1 Department of Internal Medicine, Faculty of Medicine, Ankara University, Ankara, Turkey; 2 Department of Medical Oncology, Faculty of Medicine, Cebeci Hospital, Ankara University, 06590 Cebeci/Ankara, Turkey; 3 Cancer Research Institute, Ankara University, Ankara, Turkey; 4 Medical Oncology Department, Dr. Abdurrahman Yurtaslan Ankara Oncology Education and Research Hospital, University of Health Sciences, Ankara, Turkey; 5 Department of Medical Oncology, Bakirköy Dr. Sadi Konuk Training and Research Hospital, University of Health Sciences, Istanbul, Turkey; 6 Department of Medical Oncology, Gülhane Education and Research Hospital, University of Health Sciences, Ankara, Turkey; 7 Department of Medical Oncology, Faculty of Medicine, Ankara Yıldırım Beyazıt University, Ankara, Turkey; 8 Department of Medical Oncology, Faculty of Medicine, Marmara University, Istanbul, Turkey; 9 Division of Medical Oncology, Cerrahpaşa Faculty of Medicine, İstanbul University-Cerrahpaşa, Istanbul, Turkey; 10 Department of Medical Oncology, Faculty of Medicine, İzmir University of Economics, Izmir, Turkey; 11 Department of Medical Oncology, Ankara City Hospital, University of Health Sciences, Ankara, Turkey; 12 Department of Medical Oncology, Faculty of Medicine, Trakya University, Edirne, Turkey; 13 Department of Medical Oncology, Faculty of Medicine, Gaziantep University, Gaziantep, Turkey; 14 Department of Medical Oncology, Faculty of Medicine, Hacettepe University, Ankara, Turkey; 15 Department of Medical Oncology, Faculty of Medicine, İstinye University, Istanbul, Turkey; 16 Department of Medical Oncology, Prof. Dr. Cemil Taşçıoğlu City Hospital, University of Health Sciences, Istanbul, Turkey

**Keywords:** Cancer, Tumour biomarkers, Urological cancer

## Abstract

This study aims to investigate the prognostic value of the systemic immune-inflammation index (SII)and its impact on survival in patients with metastatic renal cell carcinoma (mRCC). A total of 706patients with mRCC treated with tyrosine kinase inhibitors (TKIs)between January 2007 and June 2020 (i.e., sunitinib, pazopanib) were included in this study. SII was calculated in 621 patients with the following formula:[neutrophil (cellsx10^9^/L) x platelet (cellsx10^9^/L)] / lymphocyte (cellsx10^9^/L).All patients were classified into SII-high and SII-low groups based on the cut-off value of SII at 756, which was the median SII level of our study group. The minimal follow-up duration was 10 months in all cohorts. The median age of patients was 60 (interquartile range (IQR):53–67) years. Three out of four patients were male. The majority of patients (85.7%) had clear cell histology, and sarcomatoid differentiation was observed in 16.9% of all patients. There were 311 and 310 patients in the SII-low and SII-high groups, respectively. In general, baseline characteristics were similar in each group. However, the rate of patients treated with sunitinib (63.3% vs. 49.0%, *p* < 0.001) and those who underwent nephrectomy (83.6% vs. 64.2%, *p* < 0.001) was higher in the SII-low group than in the SII-high group. On the other hand, patients with the IMDC poorrisk (31.6% vs. 8.0%, *p* < 0.001), those with bone (51.8% vs. 32.2%, *p* < 0.001) or central nervous system (12.9% vs. 5.8%, *p* = 0.026) metastasis, and those with Eastern Cooperative Oncology Group(ECOG) 2–4 performance score (28.1% vs.17.7%, *p* = 0.002) were more common in the SII-high group than in the SII-low group. The median overall survival (OS) was longer in the SII-low group than in the SII-high group (34.6 months vs. 14.5 months, *p* < 0.001). Similarly, the median progression-free survival (PFS) was longer in the SII-low group than in the SII-high group (18.0 months vs. 7.7 months, *p* < 0.001).In multivariableanalysis, SII was an independent prognostic factor for OS (hazard ratio (HR):1.39, 95% confidence interval (CI):1.05–1.85, *p* = 0.01) and PFS (HR:1.60, 95% CI:1.24–2.05, *p* < 0.001).Pre-treatment level of high SII might be considered a predictor of poor prognosisin patients with mRCC treated with TKIs.

## Introduction

Renal cell carcinoma (RCC)accounts for 90–95% of all kidney cancers. In 2020, about 3% of all adult malignancies with an estimated 431,288new RCC cases were observed across the world^1,2^.More than 30% ofpatients diagnosed with RCC need systemic therapy for metastatic disease^3^.In the last decade, huge improvements have been observed in the mRCC treatment.Thus, immune checkpoint inhibitor (ICI) plus tyrosine kinase inhibitor (TKI)or ICI plus ICI combinations improved survival in patients with metastatic RCC (mRCC)^4,5^.

In parallel to the improvements in the treatment of mRCC, prognostic risk toolsbecame essential duringthe decision-making process in the treatment of mRCC patients. Thus, the International Metastatic RCC Database (IMDC)risk model is the standard for prognostic stratificationofpatients with mRCC treated with targeted therapies or ICIs^6,7^. The IMDC risk score is calculated by the following six parameters: Karnofsky performance status, time from diagnosis to the first systemic treatment, hemoglobin concentration, neutrophils,platelets, and corrected calcium levels. Althoughthe IMDC is a commonly usedprognostic scoring system, efforts to find a novel scoring system with fewer parameters are still continuing. Inflammatory-related peripheral cells (e.g., neutrophils, lymphocytes, platelets) derived from the peripheral blood were associated with tumor progression in various tumors. The prognostic significance of inflammatory cell parameters, such as neutrophil–lymphocyte ratio (NLR), platelet-lymphocyte ratio (PLR), C-reactive protein/albumin ratio, and systemic immune inflammation index (SII), were examined in many cancer types over the last ten years^8–15^. SIIis a combination based on the peripheral lymphocyte, neutrophil, and platelet counts. After Hu et al.showed its prognostic value in 2014, many studies established that SII couldbe a good prognostic marker in many cancer types^8^.

In this retrospective analysis, we aimed to evaluate the prognostic significance of SII in patients with mRCC treated with TKIs.

## Methods

The local ethical committee (Ankara University Faculty of Medicine Human Research Ethics Committee, approval number: 01-79-19)approved this retrospective cohort study. Informed consent was waived by “Ankara University Faculty of Medicine Human Research Ethics Committee” due to the retrospective nature of the study. This study was conducted in compliance with the “*Declaration of Helsinki”*.

### Patient population and data extraction

The Turkish Oncology Group Kidney Cancer Consortium (TKCC) database consists of approximately 1,000 patients aged 18 years and older with mRCC from 13 cancer centers in Turkey. Patientswith mRCC treated with sunitinib or pazopanib in the first-line setting were extracted from the TKCC database. Patients treated with TKIs between January 2007 and June 2020 were included in the study. The minimum follow-up duration in all patients was 10 months.

Demographic data (e.g., date of birth, gender, comorbidities, medications), date of diagnosis with RCC, the initial date of systemic treatment in the metastatic setting, Eastern Cooperative Oncology Group (ECOG) performance score, laboratory findings (e.g., neutrophil, platelet, lymphocyte counts, hemoglobin concentration, corrected calcium level),start and end dates of TKIs, and dates of progression and death were extracted from the TKCC database.

SII was calculated by using the following formula:[neutrophil (cellsx10^9^/L) x platelet (cellsx10^9^/L)] / lymphocyte (cellsx10^9^/L). All values were obtained from a complete blood count (CBC) up to 30 days before the first dose of TKIs. If there were more than one CBC result, the closest one to the initiation of TKI was used. The best cut-off value for SII was determined by using the median value of 756.In this regard, patients were divided into two groups: SII-high (≥ 756) and SII-low (< 756). The primary outcome was overall survival (OS), and the secondary outcome was progression-free survival (PFS).

### Statistical analyses

To summarize data, median with interquartile range (IQR) or mean with standard deviationand percentages were used for continuous and categorical variables, respectively. The *independent sample t-test* or *Mann–Whitney U*and *chi-square*tests were performed to compare continuous and categorical variables, respectively. Survival curves were estimated using the Kaplan–Meier method, and the differences between groups were analyzed by using thelog-rank test. Cox proportional hazards regression model was used for multivariable analyses of parameters associated with OS and PFS.OS was calculated from the initial date of TKIs to death.PFS was calculated from the initial date of TKIsto disease progression or death. Hazard ratio (HR) and 95% confidence interval (CI) were used to describe the risk factors.Harrell’s concordance index (C-index) was calculated to compare the predictive value of SII and the IMDC risk scores for OS and PFS. Differences were considered significant if the p-value was less than 0.05.All statistical analyses were performed using the SPSS 27.0 for Mac (IBM Corp., Armonk, NY).

## Results

### Baseline characteristics

A total of 706 patientswith mRCC were included in this study and SII was calculated in 621 patients. The median age of patients was 60 (IQR: 53–67) years. Three out of four patients were male.Most patients (85.7%) had clear cell histology,and 16.9%of all patients had sarcomatoid differentiation.The ECOG PS was 0 or 1 in most patients (83.5%). Approximately one out of four patients were in the IMDC poor-risk group.404 (57.2%) and 302 (42.8%)patients were treated with sunitinib and pazopanib,respectively.Approximately half of the patientsreceived interferon before TKI treatment.About three out of four patients underwent nephrectomybeforestarting systemic treatment. The lung was the most common metastatic site (51.4%).

There were 311 and 310 patients in the SII-low and SII-high groups, respectively. The rate of patients who underwent nephrectomy was higher in the SII-low group than in the SII-high group (83.9% vs. 64.4%, *p* < 0.001). Similarly, the rate of patients treated with sunitinib was higher in the SII-low group than in the SII-high group (63.3% vs. 49.0%, *p* < 0.001).The IMDC poor-risk patients’ rate was higher in the SII-high group than in the SII-low group (34.6% vs. 8.8%, *p* < 0.001). Allbaseline characteristics of the included patients are shown in Table 1.

**Table 1:** Baseline characteristics.

	All patients		SII-low patients		SII-high patients		*p*
	n = 706	(%)	n = 311	(%)	n = 310	(%)	
Age-years, median (IQR)	60 (53–67)		60 (53–69)		60 (53–70)		0.710
**Sex**							0.317
Male	531	75.2	229	73.6	239	77.1	
Female	175	24.8	82	26.4	71	22.9	
**Histological Type**							0.196
Clear Cell	563	79.7	241	77.5	257	82.9	
Non-clear Cell	94	13.3	46	14.8	36	11.6	
Missing	49	6.9	24	7.7	17	5.5	
**Sarcomatoid Feature**							0.830
Yes	83	11.8	35	11.3	39	12.6	
No	407	57.6	182	58.5	192	61.9	
Missing	216	30.6	94	30.2	79	25.5	
**Fuhrman Grade**							0.076
1–2	124	17.6	63	20.3	43	13.9	
3–4	297	42.1	129	41.4	133	42.9	
Missing	285	40.4	119	38.3	134	43.2	
**Previous Nephrectomy**							** < 0.001**
Yes	525	74.4	260	83.6	199	64.2	
No	177	25.1	50	16.1	110	35.5	
Missing	4	0.6	1	0.3	1	0.3	
**Systemic Treatment**							** < 0.001**
Sunitinib	404	57.2	197	63.3	152	49.0	
Pazopanib	302	42.8	114	36.7	158	51.0	
**IMDC Risk**							** < 0.001**
Favorable	116	16.4	83	26.7	33	10.6	
Intermediate	332	47.0	175	56.3	152	49.0	
Poor	128	18.1	25	8.0	98	31.6	
Missing	130	18.4	28	9.0	27	8.7	
**MSKCC Risk**							** < 0.001**
Favorable	91	12.9	64	20.6	27	8.7	
Intermediate	279	39.5	148	47.6	128	41.3	
High	87	12.3	27	8.7	59	19.0	
Missing	249	35.3	72	23.2	96	31.0	
**Previous Cytokine Use**							**0.032**
Yes	334	47.3	152	48.9	125	40.3	
No	372	52.7	159	51.1	185	59.7	
**Metastatic Sites**							
Lung	319	51.4	161	51.8	158	51.0	0.886
Bone	259	41.7	100	32.2	159	51.3	** < 0.001**
Liver	92	14.8	42	13.5	50	16.1	0.252
CNS	58	9.3	18	5.8	40	12.9	**0.026**
**Performance Status**							**0.002**
ECOG 0–1	515	72.9	243	78.1	207	66.8	
ECOG 2–3-4	149	21.1	55	17.7	87	28.1	
Missing	42	5.9	13	4.2	16	5.2	

*ECOG* eastern cooperative oncology group*, IMDC* international metastatic renal cell carcinoma database consortium*, IQR* interquartile range*, MSKCC* memorial sloan kettering cancer center.

Significant values are in bold.

### Survival outcomes

**Figure 1:**
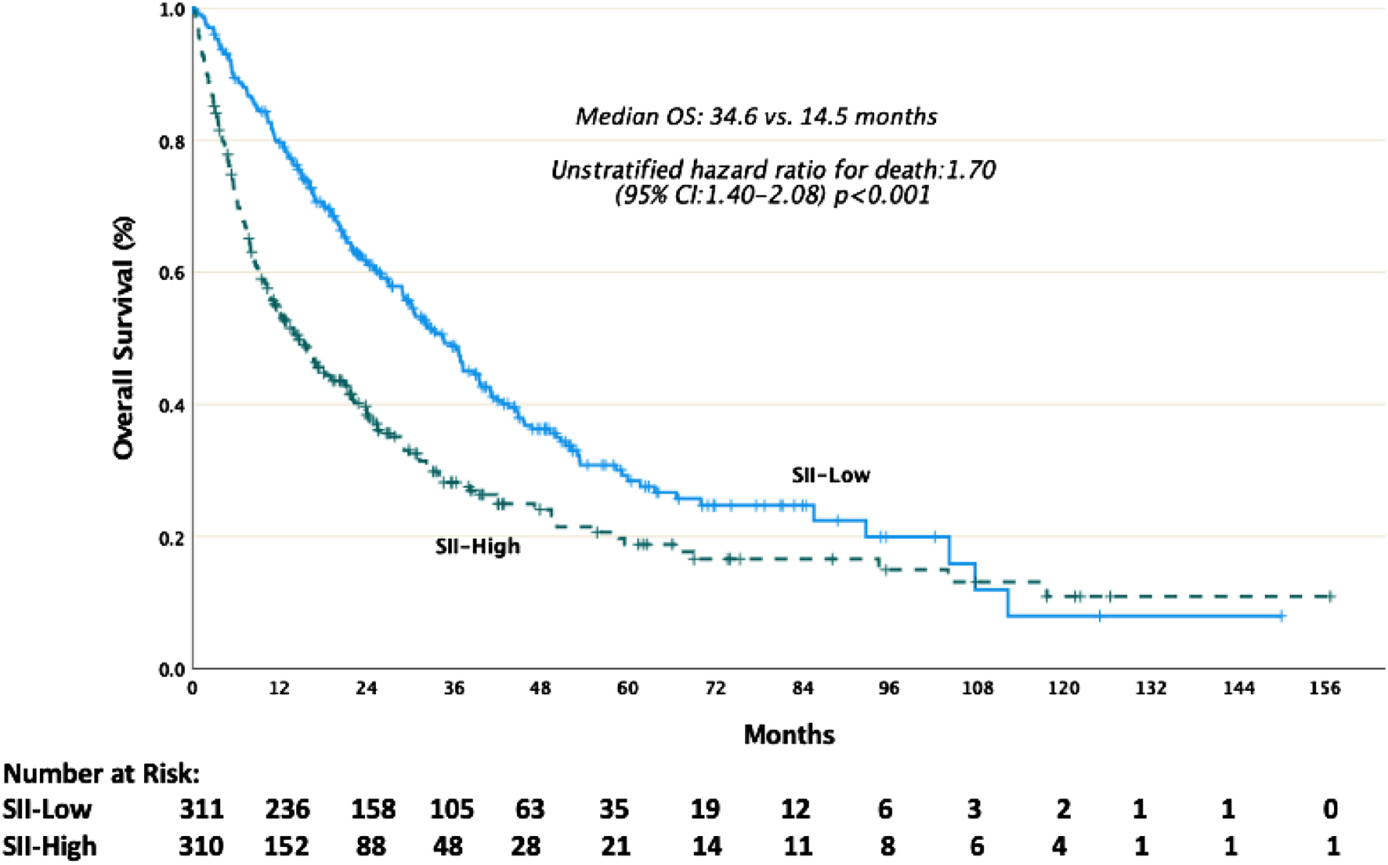
Kaplan-Meier estimates of overall survival (OS).* SII*  systemic immune inflammation index.

**Figure 2:**
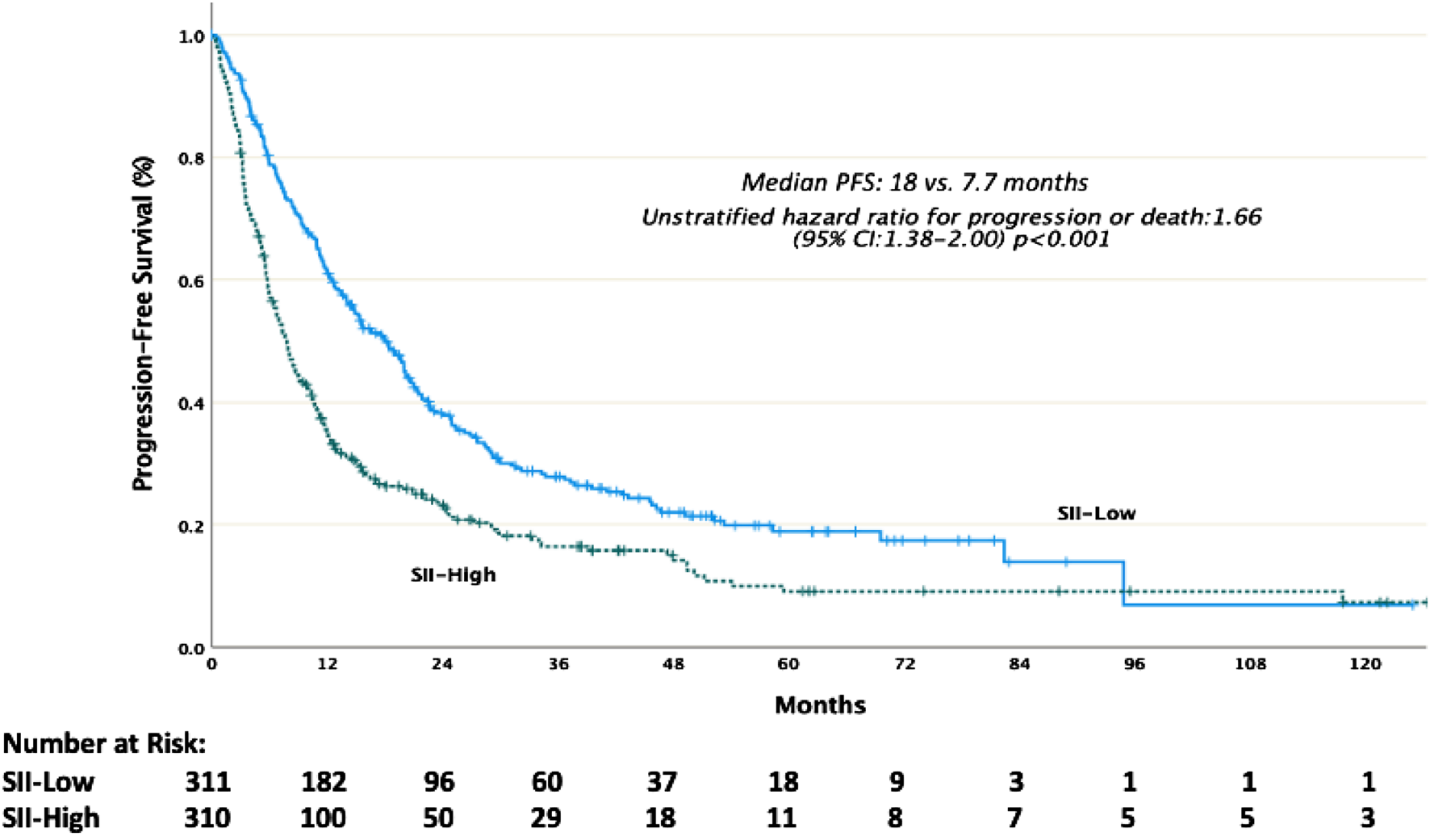
Kaplan-Meier estimates of progression-free survival (PFS). *SII*   systemic immune inflammation index.

At the median follow-up of 48.6 months, the median OS and PFS were26.1 months (95% CI: 22.5–29.7) and 11.9 months (95% CI: 10.5–13.3), respectively.The median OS was longer in the SII-low group than in the SII-high group (34.6 months vs. 14.5 months, *p* < 0.001). Similarly, the median PFS was longer in the SII-low group than in the SII-high group (18.0 months vs. 7.7 months, *p* < 0.001). Kaplan–Meier estimates of OS and PFS are shown in Figs. 1,2

After adjusting for confounding factors (age, sarcomatoid feature, nephrectomy, systemic treatment with sunitinib or pazopanib, anemia, hypercalcemia, LDH elevation, ECOG PS, time from diagnosis to systemic treatment, the total number of systemic treatment (except for IFN), and presence of bone or central nervous system (CNS) metastasis for OS; sarcomatoid feature, nephrectomy, anemia, hypercalcemia, LDH elevation, ECOG PS, time from diagnosis to systemic treatment, and presence of bone or CNS metastasis for PFS), SII was an independent prognostic factor for OS (HR:1.41, 95% CI: 1.06–1.87, *p* = 0.018) and PFS (HR:1.64, 95% CI:1.28–2.10, *p* < 0.001).Uni-and multivariable analyses of OS and PFS are shown in Tables 2, 3.

**Table 2:** Univariable and multivariable analysis of overall survival.

	Univariable			Multivariable		
	hazard ratio	95% CI	*p*	hazard ratio	95% CI	*p*
**Age**			**0.003**			0.141
< 65	1			1		
≥ 65	1.34	1.10–1.63		1.23	0.93–1.64	
**Sarcomatoid Feature**			**0.018**			**0.003**
No	1			1		
Yes	1.41	1.06–1.89		1.72	1.21–2.46	
**Nephrectomy**			** < 0.001**			**0.018**
No	1.94	1.58–2.39		1.53	1.08–2.22	
Yes	1			1		
**Systemic Treatment**			**0.034**			0.704
Sunitinib	1			1		
Pazopanib	1.22	1.01–1.48		1.05	0.79–1.39	
**Anemia**			** < 0.001**			**0.014**
No	1			1		
Yes	1.93	1.59–2.35		1.41	1.07–1.86	
**Hypercalcemia**			** < 0.001**			0.894
No	1			1		
Yes	2.21	1.52–3.21		1.04	0.53–2.06	
**LDH Elevation**			** < 0.001**			0.190
No	1			1		
Yes	1.97	1.47–2.64		1.32	0.87–2.00	
**ECOG Performance Score**			** < 0.001**			** < 0.001**
ECOG 0–1	1			1		
ECOG 2–3-4	3.18	2.58–3.91		2.75	2.04–3.71	
**Time to Systemic Treatment**			** < 0.001**			0.411
< 1 year	1.59	1.30–1.96		1.14	0.82–1.59	
≥ 1 year	1			1		
**Previous Cytokine Use**			0.491			
No	1					
Yes	1.06	0.88–1.28				
**Bone or CNS Metastasis**			** < 0.001**			0.141
No	1			1		
Yes	1.60	1.28–2.00		1.23	0.93–1.64	
Number of Systemic Treatment*			0.061			0.289
1	1.19	0.99–1.44		1.16	0.88–1.52	
> 1	1				1	
**SII**			** < 0.001**			**0.018**
Low	1			1		
High	1.70	1.40–2.08		1.41	1.06–1.87	

*CI* confidence interval*, CNS* central nervous system*, ECOG* eastern cooperative oncology group*, LDH* lactate dehydrogenase*, SII* systemic immune-inflammation index.

Significant values are in bold.

*Except for interferon.

**Table 3:** Univariable and multivariable analysis of progression-free survival.

	Univariable			Multivariable		
	hazard ratio	95% CI	*p*	hazard ratio	95% CI	*p*
**Age**			0.116			
< 65	1					
≥ 65	1.15	0.96–1.37				
**Sarcomatoid Feature**			**0.006**			**0.013**
No	1			1		
Yes	1.45	1.11–1.89		1.49	1.08–2.04	
**Nephrectomy**			** < 0.001**			0.142
No	1.77	1.46–2.14		1.26	0.92–1.73	
Yes	1			1		
**Systemic Treatment**			0.289			
Sunitinib	1					
Pazopanib	0.91	0.76–1.08				
**Anemia**			** < 0.001**			**0.008**
No	1			1		
Yes	1.65	1.38–1.98		1.39	1.09–1.79	
**Hypercalcemia**			**0.001**			0.565
No	1			1		
Yes	1.79	1.25–2.57		1.15	0.70–1.91	
**LDH Elevation**			**0.010**			0.848
No	1			1		
Yes	1.44	1.09–1.91		1.04	0.64–1.70	
**ECOG Performance Score**			** < 0.001**			** < 0.001**
ECOG 0–1	1			1		
ECOG 2–3-4	2.24	1.84–2.74		1.82	1.37–2.41	
**Time to Systemic Treatment**			** < 0.001**			0.937
< 1 year	1.51	1.26–1.82		1.01	0.75–1.34	
≥ 1 year	1			1		
**Previous Cytokine Use**			0.567			
No	1					
Yes	1.05	0.88–1.24				
**Bone or CNS Metastasis**			** < 0.001**			0.552
No	1			1		
Yes	1.48	1.25–1.75		1.07	0.84–1.38	
**SII**			** < 0.001**			** < 0.001**
Low	1			1		
High	1.66	1.38–2.00		1.64	1.28–2.10	

*CI* confidence interval*, CNS* central nervous system*, ECOG* eastern cooperative oncology group*, LDH* lactate dehydrogenase*, SII* systemic immune-inflammation index.

Significant values are in bold.

In the subgroup analysis of patients who were not treated with IFN, the median OS was longer in the SII-low group than in the SII-high group (36.4 months vs. 16.6 months, *p* = 0.001 in patients previously untreated with interferon). Similarly, the median PFS was also longer in the SII-low group than in the SII-high group(19.7 months vs. 8.1 months, *p* < 0.001) (Figures S1 and S2).

Harrell’s C-index with SII, IMDC, and MSKCC risk scoreswas 0.60, 0.63, 0.63for OS, and 0.59, 0.60, 0.61 for PFS, respectively.

## Discussion

In this multicenter study,we investigated the prognostic value of SII in patients with mRCC treated with TKIs. To the best of our knowledge, our study has the largest number of patients among studies examining the relationship between SII and survival outcomes in patients with mRCC^16–20^. The results showed that low (< 756) and high(≥ 756) SII levels had a statistically significant difference in terms of OS and PFS. Thus, SII might have a prognostic value in patients with mRCC treated with TKIs.

Many previous studies have widely investigated the relationship between inflammation and cancer. Inflammatory cells (e.g., neutrophils, macrophages, lymphocytes ) and cytokines are effective in transformation, proliferation, and metastasis in all tumor stages^21^. Neutrophils can secrete cytokines related to the stimulation of the tumor microenvironment and have a tumor-promoting activity, including cancer cell survival and proliferation, angiogenesis, and metastasis^13^. Conversely, lymphocytes inhibit tumor cell proliferation by secreting cytokines.On the other hand, platelets regulate cancer invasion, migration, and angiogenesis by secretion of numerous chemokines and growth factors ^22^. In 2014, Hu et al. developed SII to predict the prognosis of patients who underwent curative resection for hepatocellular carcinoma and established that a high SII score (> 330 × 10^9^ cells/L) indicated a poor outcome for those patients^8^. Subsequently, SII has been investigated as a marker to predict cancer survival in various tumors, such as gastric cancer, germ-cell tumor, and prostate cancer^6–12^^.^. A recentstudy that evaluated the impact of SII on the survival of patients with mRCC treated with TKI was published in 2020. In this study, Teishima et al. showed that low SII was associated with poorer survival in 179 patients with mRCC treated with TKI^16^. Furthermore, the pre-treatment SII cut-off value was determined as 730 × 10^9^ cells/L in the study of Teishima et al., which was numerically close to SII cut-off value of our study. It should be noted that the number of patients was higher in our study.Another study that investigated the relationship between SII and survival in patients with mRCC treated with TKIs was reported by Lolli et al. They included 335 patients with mRCC and concluded that pretreatment SII was an independent prognostic factor OS^12^.

In addition to prognostic value in patients with mRCC, SII was also evaluated as a prognostic marker inearly RCC patients. The studies concluded that SII was an accurate prognostic marker irrespective of disease stage in RCC^19,20^. Actually, this result may be associated with the role of immune system in the clinical course of RCC irrespective of clinical stage^23^.

We have several approved prognostic scoring systemsin patients with mRCC. The IMDC and Memorial Sloan Kettering Cancer Center (MSKCC) were the most popularrisk models^24,25^.However, the IMDC and MSKCC risk scores are calculated by using six and five parameters, respectively. In our study, C-index values were almost similar for OS and PFS in SII, IMDC, and MSKCC risk scores. SII could provide the same prognostic accuracy as the IMDC and MSKCC, despite only including neutrophils, platelets, and lymphocytes in the equation.About the prognostic value of IMDC risk score and SII combination, Chrom et al.showed thatreplacement of neutrophil and platelet counts with SII in the IMDC risk model increased the accuracy of the IMDC risk model.It should be noticed that they also used a cut-off value of 730 × 10^9^ cells/L for SII, which is almost the same asour study^26^.Furthermore, as a result of efforts to find a novel prognostic marker in patients with mRCC, Başal et al. showed that SII could predict survival in each IMDC risk group^27^.

Our survival results were also compatible with the pivotal study of sunitinib,including previously untreated patients with mRCC. They reported that the median OS was 26.4 months and PFS was 11 months in patients with mRCC receiving sunitinib, which was also numerically close to our study’s survival results^28^.

Our study has several limitations due to its retrospective nature. First, we had a lack of data to calculate SII in some patients. Because of this reason, we had to exclude those patients from our study. Second,the time interval betweenobtaining laboratory values to calculate SII and the initial date of TKIs might be different in each included center.Third, mRCC patients treated with interferon before TKI treatment were included in our study. ICI plus TKI or ICI plus ICI combinationsare accepted as the standard of care in the first-line treatment of patients with mRCC. Althoughcombinationsare considered standard treatment, there is still a subgroup of patients who benefit from TKI alone. ICI plus TKI studies concluded that no clear difference between the sunitinib and combination arms in survival outcomes in the IMDC favorable risk group^29–31^. All these findings suggest that we cannot completely abandon TKIs in the treatment of patients with mRCC.

In conclusion, our study showed the prognostic value of SII in mRCC patients treated with TKIs. In this context, SII, an easily accessible marker, might lead to establishing novel therapeutic strategies or risk models in patients with mRCC treated with TKIs.Although -studies evaluated prognostic effect of SII on patients treated with ICI,the relationship of ICIs plus TKIs combinations with SII has not been investigated yet^32,33^. SII may be a potential prognostic marker for RCC patients treated with ICI and TKIs combination from a future perspective.

## Supplementary Information

Supplementary Information.
