# Supplementary material for: The relationship between systemic immune inflammation index and survival in patients with metastatic renal cell carcinomatreated withtyrosine kinase inhibitors
Source: Sci Rep. 2022 Oct 3;12:16559. doi: 10.1038/s41598-022-20056-3 (PMC9529965; doi:10.1038/s41598-022-20056-3)

**The Relationship Between Systemic Immune Inflammation Index and Survival in Patients with Metastatic Renal Cell Carcinoma Treated with Tyrosine Kinase Inhibitors**

Kadriye Bir Yücel^1^, Emre Yekedüz^2,3^, Serdar Karakaya^4^, Deniz Tural^5^, İsmail Ertürk^6^, Cihan Erol^7^, Özlem Ercelep^8^, Nihan Şentürk Öztaş^9^, Çağatay Arslan^10^, Gökhan Uçar^11^, Ahmet Küçükarda^12^, Özlem Nuray Sever^13^, Saadettin Kılıçkap^14,15^, Orçun Can^16^, Satı Coşkun Yazgan^1^, Berna Öksüzoğlu^4^, Nuri Karadurmuş^6^, Mehmet Ali Şendur^7^, Yüksel Ürün^2,3^

1. Ankara University Faculty of Medicine Department of Internal Medicine, Ankara, TURKEY
2. Ankara University Faculty of Medicine Department of Medical Oncology, Ankara, TURKEY
3. Ankara University Cancer Research Institute, Ankara, TURKEY
4. University of Health Sciences Dr. Abdurrahman Yurtaslan Ankara Oncology Education and Research Hospital, Medical Oncology Department, Ankara, TURKEY
5. University of Health Sciences, Bakirköy Dr. Sadi Konuk Training and Research Hospital, Department of Medical Oncology, İstanbul, TURKEY
6. University of Health Sciences, Gülhane Education and Research Hospital, Department of Medical Oncology, Ankara, TURKEY
7. Ankara Yıldırım Beyazıt University, Faculty of Medicine, Department of Medical Oncology, Ankara, TURKEY
8. Marmara University, Faculty of Medicine, Department of Medical Oncology, İstanbul, TURKEY
9. İstanbul University-Cerrahpaşa, Cerrahpaşa Faculty of Medicine, Division of Medical Oncology, İstanbul, TURKEY
10. İzmir University of Economics, Faculty of Medicine, Department of Medical Oncology, İzmir, TURKEY
11. University of Health Sciences, Ankara City Hospital, Department of Medical Oncology, Ankara, TURKEY
12. Trakya University, Faculty of Medicine, Department of Medical Oncology, Edirne, TURKEY
13. Gaziantep University, Faculty of Medicine, Department of Medical Oncology, Gaziantep, TURKEY
14. Hacettepe University, Faculty of Medicine, Department of Medical Oncology, Ankara, TURKEY
15. İstinye University, Faculty of Medicine, Department of Medical Oncology, İstanbul, TURKEY
16. University of Health Sciences, Prof. Dr. Cemil Taşçıoğlu City Hospital, Department of Medical Oncology, İstanbul, TURKEY


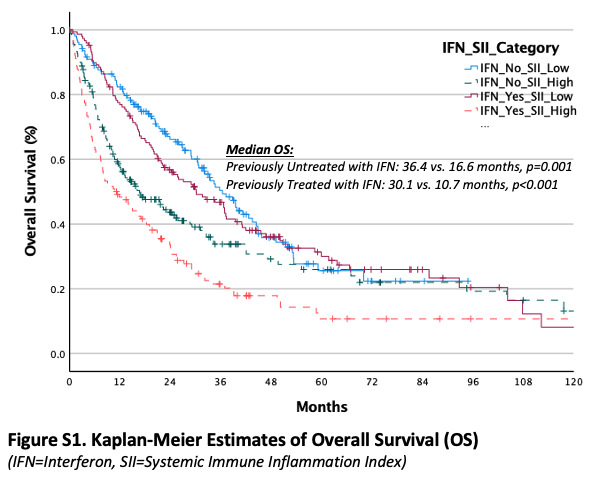


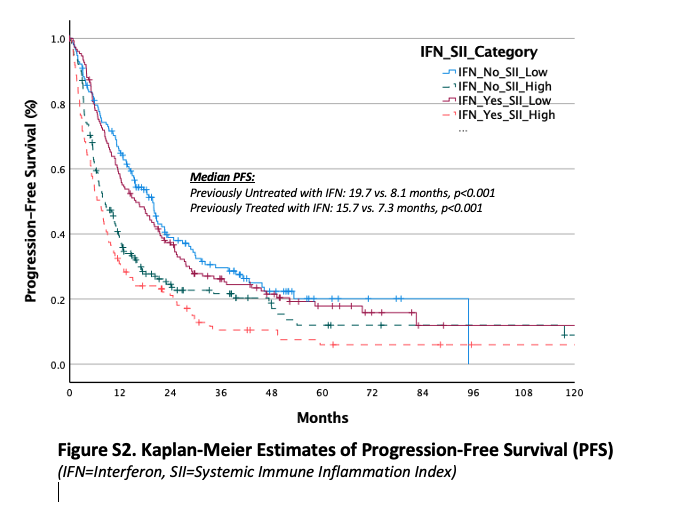

Supplement: Supplementary file 1 — Supplementary Information. [file 41598_2022_20056_MOESM1_ESM.docx]
